# Enviro-economic and optimal hybrid energy system: Photovoltaic–biogas–hydro–battery system in rural areas of Pakistan

**DOI:** 10.1016/j.heliyon.2024.e35182

**Published:** 2024-07-30

**Authors:** Safyan Mukhtar, Shakoor Muhammad, Haifa A. Alyousef, Wajid Khan, Rasool Shah, Samir A. El-Tantawy

**Affiliations:** a Department of Basic Sciences, Preparatory Year Deanship, King Faisal University, Al-Ahsa, 31982, Saudi Arabia; b Department of Mathematics and Statistics, College of Science, King Faisal University, Al-Ahsa, 31982, Saudi Arabia; c Department of Mathematics, Abdul Wali Khan University Mardan, Khyber Pakhtunkhwa, Pakistan; d Department of Physics, College of Science, Princess Nourah bint Abdulrahman University, P.O. Box 84428, Riyadh 11671, Saudi Arabia; e Department of Computer Science and Mathematics, Lebanese American University, Beirut, Lebanon; f Department of Physics, Faculty of Science, Port Said University, Port Said 42521, Egypt; g Department of Physics, Faculty of Science, Al-Baha University, Al-Baha, P.O. Box 1988, Saudi Arabia

**Keywords:** Photovoltaic, Hydropower, Biogas, Bettry energy system, HOMER, Energy management, Optimization

## Abstract

To satisfy the electricity needs of a village in Tangi, northwest Pakistan, the present research can design and evaluate the environmental and economical aspects of an optimal hybrid photovoltaic-biogas-hydropower-battery energy sustainable system (PV-BG-HP-BESS). This framework integrates various renewable energy sources, delivering a modern, efficient approach to sustainable energy solutions. The HOMER Pro software is utilized to optimize the most economical and effective hybrid energy system. The results showed that the proposed hybrid system comprising 91.4 kWp PV modules, 19.6 kW hydropower, a 50 kW biogas generator (BG), 36 batteries, and a 60.6 kW converter was the most economical choice. This system, which used the cyclic charging (CC) method, had a cost of energy (COE) of 0.0728 $/kWh and a total net present cost (NPC) of $152,242. The suggested hybrid energy system for rural areas of Pakistan includes photovoltaic (PV), biogas (BG), hydro, and battery components to provide a dependable and sustainable power supply. This system minimizes the need for expensive fossil fuels while simultaneously minimizing environmental impact by lowering pollutants and greenhouse gas emissions. The system's annual electricity production is 294,782 kWh, with PV leads at 59.4%, BG at 6.02%, and hydro at 34.6%, ensuring uninterrupted power generation even in remote areas. The unmet load, extra electricity, and capacity shortage illustrate the reliability of the system and make it possible to address rural electrification challenges while supporting sustainable development and economic growth. Moreover, the outcomes of the proposed hybrid system dominate the previous studies in multiple objectives, including cost and sensitivity analysis, when compared.

## 1 Introduction

Reliable and continuous electrical supply is essential in Khyber Pakhtunkhwa, Pakistan's rural areas. In order to create an environmentally friendly and highly efficient hybrid energy system, this research integrates technology related to solar, biogas, hydro, and batteries. To increase energy sustainability and reliability, the system provides a state-of-the-art and efficient solution to the region's energy issues. In distant rural locations, utility suppliers face substantial losses in transmission and distribution of power [1]. Because of the growing consumption of fossil fuels and the resulting environmental harm, it is crucial to create sustainable energy alternatives, such as biomass, wind, solar, and hydropower, to substitute conventional energy sources [2]. Researchers have just started a study that uses HOMER software to simulate a BG/PV/hydro hybrid system combined with biomass power generation. The outcomes revealed that the optimal configuration, comprising a 10 kW biomass generator, 5 kW pico-hydro plant, and 72 kWh battery storage, generated electricity for 0.352 /kWh tailored explicitly for meeting the energy demands of remote villages [3]. The research focuses on utilizing cattle and buffalo manure in Pakistan to generate green electricity. It emphasizes the environmental benefits of biogas production from manure, including reducing global warming and contributing to sustainable energy [4]. In Pakistan, livestock manure is an important bioresource for the production of renewable energy. Punjab has the highest concentration of livestock, followed by other provinces of Pakistan, including Sindh, Baluchistan, and KPK. Moreover, approximately 10 million households manage livestock. Animal manure from Pakistan has the capacity to generate 26,871.35 million cubic meters of biogas, 492.6 pet joules of heat energy, and 5521.5 megawatts of electricity in 2018, demonstrating its ability to help with the nation's energy problems. It is imperative to implement national programs that support home biodigesters, since the country has the ability to build 5 million biodigesters in different agricultural regions [5].

**Figure 1:**
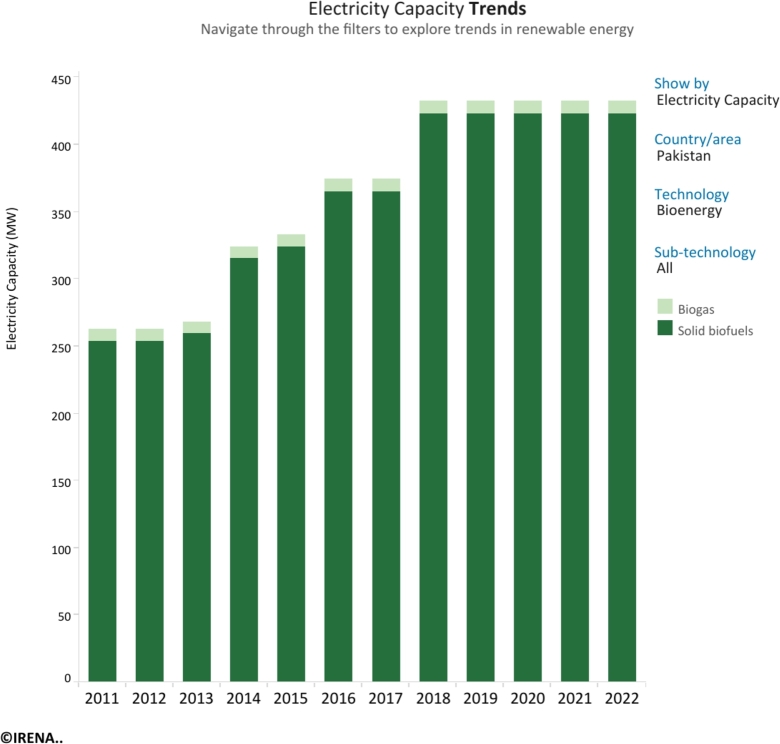
Development of bioenergy capacity in Pakistan between 2011 and 2022.

**Figure 2:**
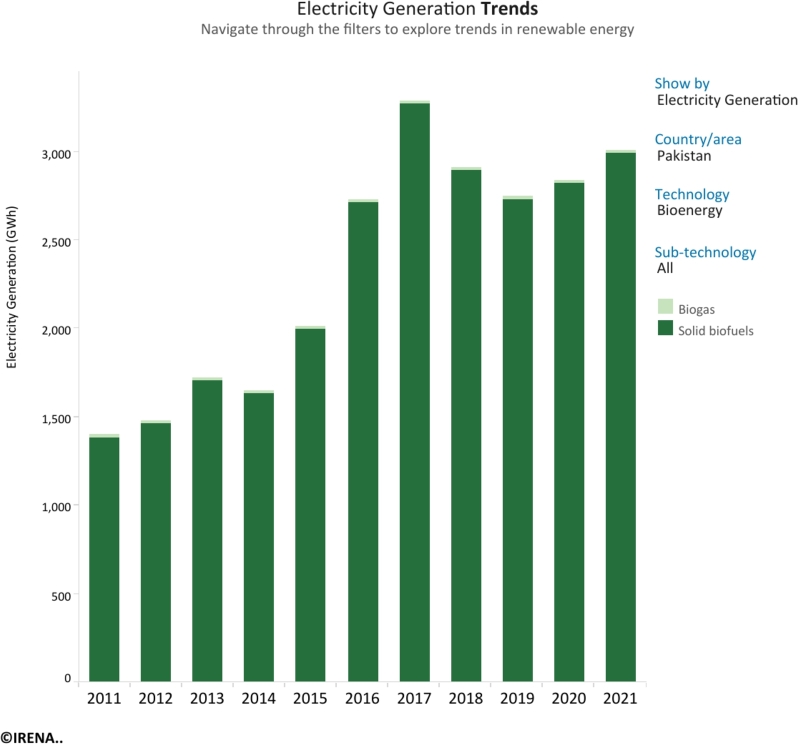
Electricity potential in Pakistan from bioenergy from 2011 to 2021.

Figs. 1 and 2 [6] underscore the evolving bioenergy landscape in Pakistan from 2010 to 2020. While the overall installed capacity has steadily increased, a notable shift is observed within specific bioenergy sources. Notably, renewable municipal waste capacity demonstrates a robust growth trajectory, indicating a promising avenue for sustainable energy utilization. On the other hand, the slower rate of increase in biogas capacity points to possible areas for additional investment and optimization. Analyzing electricity generation patterns reveals a coherent correlation with current operational capacity trends, reinforcing the significance of innovative thinking and strategic planning to maximize bioenergy's contribution to Pakistan's energy mix. Renewable energy is lauded for its sustainability and benefits for the environment. Yet, difficulties including intermittency, volatility, and poor controllability continue [7], [8]. Adopting a hybrid renewable energy approach that uses different energy sources simultaneously appears to be a strategic answer to enhance the dependability of the energy system [9].

Recent studies show researchers' interest in hybrid renewable energy systems that include biomass power generation has increased. Ngundam and Nfah [3] have simulated a hybrid system consisting of photovoltaic (PV), biomass gasification (BG) and hydro component with the detail HOMER software tool. This technique was created to satisfy the power needs of a dormitory. According to their analysis, the optimal configuration—which consisted of a 5 kW pico-hydro plant, a 72 kWh battery storage system, and a 10 kW biomass gasifier—could produce power for 0.352 /kWh, making it a practical choice for providing remote settlements with inexpensive and sustainable energy. Rahman et al. [11] assessed the feasibility of economically combining solar and biomass energy in hybrid systems using HOMER. Their results showed that families with three to six cows might effectively power their culinary and electricity needs by combining solar PV with methane. Sigarchian and colleagues are in a remote region of Kenya's Garissa district. [12] investigated the viability of a hybrid power-producing system integrating solar, wind, BG, and battery technologies using the HOMER software. Such system with backup biogas was the best option, according to their study. Power was produced at rates of 32% by biogas, 19% by wind, and 49% by PV. Mudasser et al. [13] investigated the viability of grid-connected hybrid biogas/wind systems at three distinct sites using the HOMER software. According to their analysis, most of the biogas/wind hybrid systems they looked at are not financially practical. This is mainly because the costs associated with producing renewable energy cannot be met by the community feed-in tariff currently in place, which is based on kilowatt-hours.

Sharma and Goel [14] looked at the viability from a economical and technical standpoint of a standalone hybrid energy system with a biogas (BG) component to supply power to 124 homes in a rural area of eastern India. With a carbon footprint of 10,346 kg/year, the lowest Net Present Cost (NPC) of $386,971, and a minimal Cost of Energy (COE) of $0.476/kWh, the solar-biogas generator was found to be the most practicable choice based on their study. Mishra et al. [15] designed and modeled the configuration of a biomass/wind and PV/biomass system for India using the HOMER software. According to their investigation, the hybrid PV/biomass system architecture fared better for decentralized electrification regarding environmental impact, cost-effectiveness, and reliability than the wind/biomass system. Janajreh and Ghenai [16] integrated a hybrid using the HOMER paradigm in Sharjah by including photovoltaic/biomass microgrid. Based on their research, the hybrid power production system may supply 14% of Sharjah City's annual electricity needs, with the PV system supplying 74% and the biomass gasification (BG) system providing 26%. Bhatt et al. [17] studied off-grid hybrid energy systems' economic and technological viability in rural Uttarakhand, India. They examined battery, solar, micro-hydro, diesel, and biomass combinations and battery, diesel, and biogas arrangements. Using the HOMER program, the research determined that the best configuration included micro-hydro, photovoltaics, biomass, diesel, biogas, and batteries. The overall NPC for this system was $533,654, the COE was $0.197/kWh, the RF was 94%, and the CO2 emissions were 15,930 kg annually.

Moreover, the research [44] investigated a combined solar PV-biogas-battery energy system for a commercial site in Berkane, Morocco, with a focus on its effectiveness and environmental benefits. In addition, the study utilized the HOMER software and real data to determine that the most efficient configuration consists of a 170 kW biogas generator, 231 kW PV modules, a 201 kWh Li-Ion battery, and a 140 kW converter. Compared to a system fueled only by biogas, this design results in a levelized cost of electricity (LCOE) of 0.280 $/kWh. In order to meet Babadam, northern Cameroon's energy demands, Yimen [48] evaluated and optimized two hybrid PV/wind/battery systems using different biomass energy methods with the help of HOMER software. With a cost of electricity (COE) of $0.347/kWh, the optimal system included 200 batteries, a 30 kW biogas generator, and a 98.1 kW PV module. Moreover, the combination of biogas generators lowered COE by 29% when compared to the PV/wind/battery system. Munuswamy [49] examined the expenses of fuel-cell and grid-powered power plants for a rural health facility using HOMER software. A systematic review and meta-analysis of fossil fuel and renewable energy consumption is given [45]. On similar lines, one of the studies given in [46] states that renewable energy will replace fossil fuels by 2050. For distances greater than 44 kilometers, the study discovered that off-grid systems proved more cost-effective than grid-based supply. However, it is essential to note that the study concentrated on one organization rather than a rural community everywhere. Jahangiri [10] used HOMER software to explore four hybrid systems in Zarrin Shahr, Isfahan province, that utilized solar, wind energy, and biomass for simultaneous generation and heating. Results indicated that if the study location was less than 2.58 kilometers from the national grid junction, getting electricity from the grid was better than utilizing biomass energy. In addition, the result indicated that the optimal hybrid energy BG/PV/battery system could be identified at a cost of $1.019/kWh and an overall NPC of $25121. Therefore, biomass-based systems [47] have gained acceptance from the above discussion due to their environmental advantages and lower costs. On the same lines but differently, the proposed PV-Hydro-Biogas-Bess is presented, which has a low coast and is environmentally friendly.

An ideal hybrid energy system design including solar panels, biogas generators, diesel, and batteries is presented by Li et al. [18] for a Xuzhou, China hamlet. The study finds an economical setup with a 100 kW biogas generators, 400 kWp PV array, a 200 kW converter, and 400 batteries that operates in a load-following manner using HOMER software. This system demonstrates COE of 0.24 $/kWh, a total NPC of $1,808,992, and significant annual CO2 savings of 1,297,174 kg compared to a diesel generator. Furthermore, sensitivity analysis suggests that cost reductions in biogas generators and batteries could enhance the economic viability of this hybrid solution for rural villages in developing countries.

### 1.1 Motivation

The present hybrid system is a different and optimal approach as compared to the above systems. In this study, we have considered photovoltaic, biogas, hydropower, and battery energy systems (PV-BG-HP-BESS), which result in an economically optimal and environmentally friendly hybrid energy system. This system is a location-based system in rural areas that encounters specific difficulties when trying sustainable energy solutions, such as unreliable electrical power and environmental concerns. Integrating solar, biogas, hydroelectric, and battery technologies presents multiple possibilities for successfully dealing with these issues. This study aims to develop an ideal energy mix for rural Pakistan to satisfy their specific needs via solar energy, agricultural waste biogas, and local hydropower. Combining all of these elements into a single system, we hope to provide dependable, environmentally friendly energy while supporting economic growth in these areas. This study seeks to show that integrated systems like this are both financially viable and environmentally friendly and might work as a model for other regions worldwide.

### 1.2 Objectives

This research paper aims to establish and operate an effective and sustainable hybrid energy system in rural areas of Pakistan. The system aimed at integrating photovoltaic, biogas, hydroelectric, and battery technologies to supply a reliable, over-time power source that decreases environmental impact and overall expenses. This study aims to gain knowledge of the proposed hybrid energy system's scientific, economic, and ecological components and demonstrate its effectiveness in addressing the energy needs of Pakistan's rural areas. Therefore, the key objectives of this paper can be mentioned as follows:

I. To create and supervise high-quality hybrid energy systems that interact with photovoltaic, biogas, hydro, and battery storage innovations to fulfill the specific requirements of Pakistan's rural regions' energy requirements.
II. To decrease energy waste and use it for valuable purposes through new hybrid energy usage processes.
III. To identify and implement the most effective things for hybrid energy systems to decrease total system costs involve maintenance costs.
IV. To create cleaner, more environmentally friendly power generation systems that promote clean and low-cost energy in rural areas of Pakistan.
V. To validate the proposed hybrid energy system simulation scenario using HOMER software to deliver clean energy and improve its financial viability.
VI. To increase economic growth in rural areas by encouraging the utilization of sustainable energy sources and decreasing reliance on unsustainable energy sources.
VII. To help alleviate Pakistan's energy crisis by supplying dependable, eco-friendly power sources.
VIII. To demonstrate how integrated hybrid energy systems can effectively address energy challenges and promote sustainability, serving as a blueprint for other regions worldwide.
IX. To play a vital role in reaching national and international sustainable development objectives, like the UN Sustainable Development Goals (SDGs), using hybrid energy systems.

Shahzad et al. [19] introduced a hybrid energy system merging biomass and PV to electrify off-grid rural areas in Punjab, Pakistan, specifically for irrigation needs. Their findings suggest that a configuration consisting of a 8 kW biogas generator, 10 kW PV array, 32 storage batteries, and 12 kW converter is a practical solution for this hybrid system. The implementation of this system resulted in savings of PKR 4.84 per kilowatt-hour, demonstrating its economic viability. For the BS link canal-1, M. Kamran et al. suggest a hybrid energy system that combines solar PV, run-off canal hydropower, and wind turbines. Based on the availability of renewable resources, three solutions are examined using the HOMER programme. Micro-hydro and solar combined with load shedding is the most economical approach, lowering NPC to $284,877 and COE to $0.0437/kWh. Such hybrid systems, which provide dependability and efficiency above traditional power plants, are perfect for rural electrification in Pakistan due to the country's rich renewable energy resources. Pakistan is shifting from conventional to renewable energy consumption, especially in rural regions, in response to climate change and depleting nonrenewable energy sources. However, owing to poor income, limited access to financing, education, and government backing, the adoption of biogas technology among rural families in three districts of Khyber Pakhtunkhwa Province continues to be low. The study identifies barriers and recommends boosting biogas technology adoption in rural Pakistan [31]. The development of bioenergy in recent years and the electricity potential from this source in Pakistan are given in Fig. 1 and Fig. 2, respectively.

There is a dearth of information in the literature about applying hybrid power generation systems in Pakistani rural areas that combine solar photovoltaic (PV), biomass, and hydroelectricity. Furthermore, such studies have been used in Pakistan that use biogas made from livestock manure. To integrate solar PV, biogas, batteries, and hydroelectricity into hybrid renewable energy systems in Pakistan, this article presents novel methodologies. It thoroughly examines the power generation systems' environmental and economic performance. The current work uses cutting-edge modeling techniques to investigate the economic evaluation and optimization of these hybrid sustainable energy systems in Pakistani rural areas.

Although the proposed hybrid system looks simple, extra care will be needed during its installation, particularly regarding increased water flow, which generally happens during the monsoon season and happens unexpectedly during February and March. To take into account this excess water flow without affecting the system, special considerations must be taken during installation and manufacturing. Moreover, the system is simple enough, with backups to ensure uninterrupted electricity. If one energy source fails to meet demands, an alternate supply always exists to meet the load demands. Apart from this complexity, as stated above, and to the fullest of our understanding in light of the presented literature review, the proposed system dominates the other hybrid systems in the literature in multiple ways, which are given below.

As presented in Table 1, the proposed system dominates the remaining hybrid systems in terms of energy cost per kilowatt. Also, the proposed system dominates the remaining hybrid systems regarding net present cost, as given in Table 1. Moreover, the proposed system dominates the remaining hybrid systems in terms of emissions of CO2 gases, as given in Table 1. Therefore, for all objectives, the proposed hybrid energy systems dominate the remaining similar studies in the literature.

**Table 1:** Comparison of hybrid energy system in cost of energy, total net present cost and CO2 emission.

Study	System configuration	Cost of	Totol net	CO2 emissions
		energy ($)	present cost ($)	kg/yr
Proposed approach	PV-BG-HP-BESS	0.0738	152,242	1.29
Chong [18] (2022)	PV-BG-DG-BESS	0.24	1,808,992	72,047
Jahangiri [10] (2018)	PV-BG-BESS	1.019	25,121	90
Bhatt [17] (2016)	HP-PV-BG-DG-BESS	0.197	533,654	220

This is how the rest of the article is structured: The materials and technique used for this investigation are presented in Section 2. Section 3 presents the findings and the resulting comparison. Section 4 concludes this study with the findings.

## 2 Methods and softwares

### 2.1 HOMER Pro software

Many computer based evaluation tools for the analysis of renewable energy systems are easily obtained. These include technical economy and optimization models like HOMER and iHOGA, financial assessment models like SAM-System Advisor Model, transient system simulation programmes like TRNSYS, PV simulation tools like PVsyst and SOLSIM, and simulation models like HYBRID2, HYBRIDS, and HYDROGEMS. One example of software for pre-feasibility study is the clean energy management model from RETScreen. Table 2 provides an overview of these tools and demonstrates how valuable they are for simulating energy systems. [22–31].

**Table 2:** Simulation softwares for energy systems.

Software	Water power	PV power	Free trial	Technical	Economic	Optimization
Pvsyst	No	Yes	Yes	Yes	no	Yes
HYDROGEMS	Yes	Yes	No	Yes	yes	Yes
Hybrid2	Yes	Yes	Yes	Yes	no	No
SOLSIM	No	Yes	No	Yes	yes	Yes
HYBRIDS	Yes	Yes	No	Yes	no	Yes
RETScreen	Yes	Yes	Yes	Yes	yes	No
TRNSYS	Yes	Yes	Yes	Yes	no	No
Ihoga	Yes	Yes	No	Yes	yes	Yes
HOMER	Yes	Yes	Yes	Yes	yes	Yes
SAM	Yes	Yes	Yes	Yes	yes	No

The HOMER software, created by the National Renewable Energy Laboratory (NREL), is publicly accessible software for assessing and optimizing the technical and financial viability of energy systems [20]. To mimic different systems, it uses a variety of input data, such as resource, load, technical characteristics, and more. Applying such software aims to reduce the net present cost of the energy system (NPC) [23], [24], [25], [26], [27], [28]. This procedure is shown graphically in the software's simulation diagram. HOMER is a useful tool that makes it easier to analyze and optimize energy systems thoroughly, which is important when making decisions on renewable energy projects. It is recommended to consult the official HOMER software documentation or the NREL website for the most recent features and upgrades.

**Figure 3:**
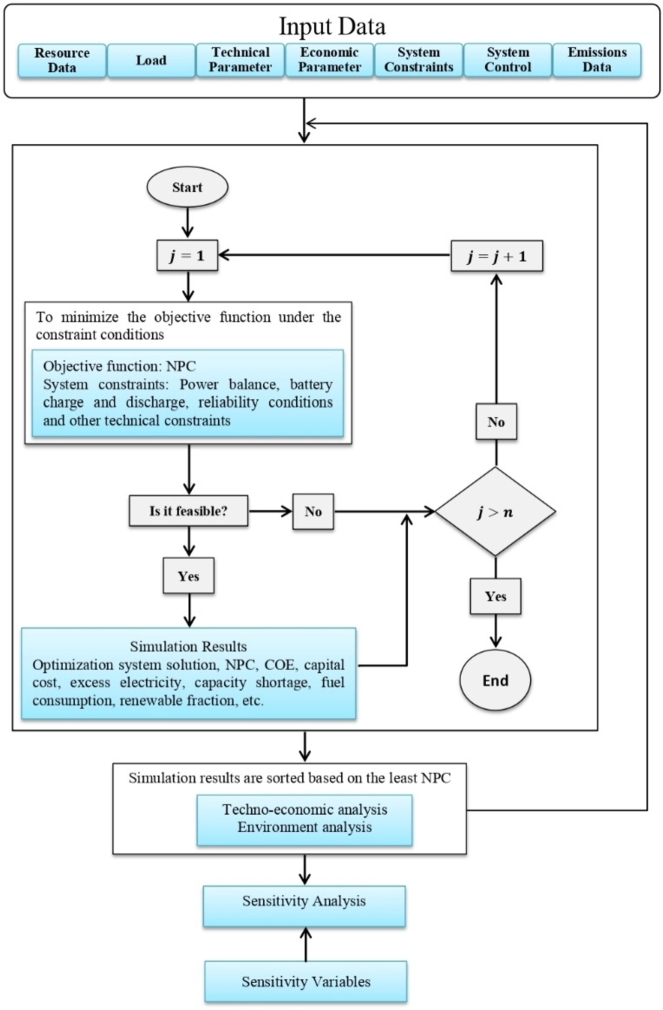
Block diagram for the HOMER software algorithm and simulation [22], [23], [24], [25], [26], [27].

Furthermore, the technical discussions on system optimization and dispatch strategy implementation are carried out using HOMER Pro software, which uses optimization algorithms to locate the most economical and effective hybrid energy system purpose in Fig. 3. The method of optimization begins with the input of different data, such as the energy sources that are accessible (such as solar, biogas, and hydropower), equipment costs, fuel prices, and the site's energy demand. The program then simulates thousands of various system arrangements by changing the size and mixture of these elements.

For each setup, the software conducts a comprehensive evaluation, calculating the total net present cost (NPC), incorporating capital costs, repair expenses, fuel costs, and any possible revenues. It also examines the system's capacity to meet energy demands regularly. By assessing these NPC values, HOMER Pro establishes the setup with the lowest cost while maintaining its necessary demand for energy, instilling confidence in the engineers, energy system designers, and scholars in the field of renewable energy.

This helps ensure that the chosen system setup is both robust and cost-effective in a number of future scenarios. Importantly, HOMER Pro's optimization algorithm aids in the development of a hybrid energy system that carefully balances cost, accuracy, and adverse environmental effects, providing reassurance to engineers, energy system designers, and researchers in the field of renewable energy.

The formula below is used to represent the total NPC [21], [22], [23], [29].

(1) $$NPC=\frac{C_{ta}}{CRF(i,N)}$$

Equation (1) represents the annual cost of a project that takes into consideration the project duration (N) and real interest rate (i) is called the total annualized cost (CTA). How the original investment is distributed throughout the course of the project is determined by the capital recovery factor (CRF (i, n)) [21], [22], [23], [29].

(2) $$CRF(i,N)=\frac{i{\left(1+i\right)}^{n}}{{\left(1+i\right)}^{n}-1}$$

The above equation (2) represents the life-cycle cost is divided by the total electrical load provided to get the cost of energy (COE), where “n” is the number of years. Over the course of the project, this ratio assesses the cost-effectiveness in relation to the amount of power used [21], [22], [23], [29].

(3) $$COE=\frac{C_{ta}}{E_{served}}$$

In equation (3) the whole quantity of electricity utilized is serviced. (kWh/year).

The energy provided to the load by renewable sources is divided by the total energy used to get the Renewable Fraction (RF). This ratio shows how much energy is used overall and how much of it is derived from renewable sources [30].

(4) $$F_{RF}=(1-\frac{E_{nr}+H_{nr}}{E_{t}+H_{t}})100$$

In equation (4) the annual nonrenewable thermal output (Hnr) is represented by kWh/yr, the annual nonrenewable electrical production (Enr) by kWh/yr, the annual total electrical load (Et) by kWh/yr, and the annual total thermal load (Ht) by kWh/yr.

### 2.2 Location of case study, solar resource availability, and load estimation

**Figure 4:**
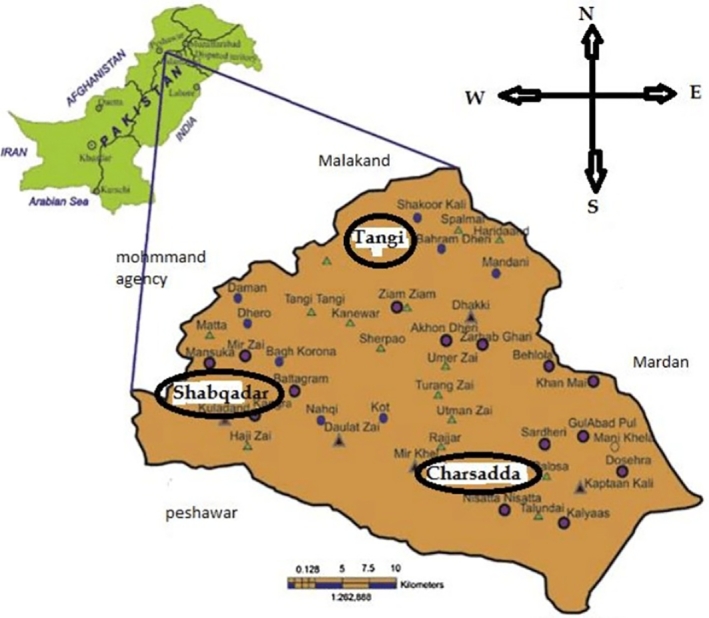
Case study location of Tangi Charsadda city, Pakistan.

**Figure 5:**
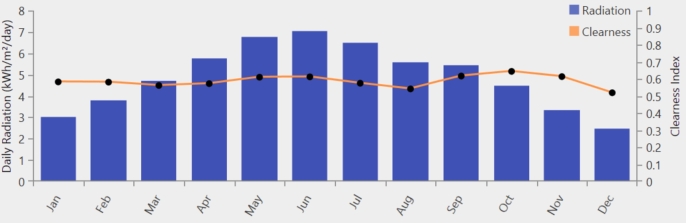
Data on the clearness index and monthly average sun radiation for Charsadda, Pakistan.

**Figure 6:**
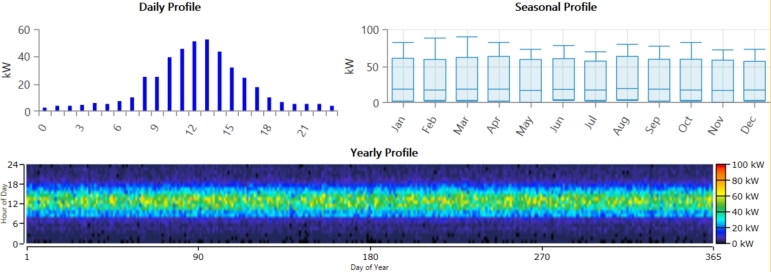
The village under consideration's hourly and monthly load profile.

Tangi is a small village in Khyber Pakhtunkhwa, Pakistan, lies between Peshawar and Mardan, near the Swat River, with coordinates (34.303955, 71.655472). Fig. 4 represents the location of the case study Tangi, district Charssada, kp, Pakistan. Tangi is situated in Charsadda, which is a 996 square kilometer region bordering to the north by Malakand, to the east by Mardan, to the south by Nowshera and Peshawar, and to the west by Mohmand. Charsadda has an excellent agricultural environment close to the Indus River [32]. With crops like sugarcane and wheat growing on its fertile soil and an abundance of water sources, especially from the Kabul River, the region's agricultural industry thrives. While sugarcane is a significant revenue crop and a staple food crop, wheat is important economically. Charsadda's agriculture mostly uses antiquated techniques like tube wells and canal irrigation [33]. A large proportion of the population is employed in agriculture, particularly in the production of wheat and sugarcane, which drives the local economy. With four different seasons with an average rainfall of about 869.9 mm, and an average temperature of around 22.2 degrees Celsius, Charsadda has a moderate temperate monsoon climate. Fig. 5 displays the solar radiation and clearness index. Table 3 displays the anticipated load demand for Tangi village throughout the winter (December to February), spring (March to May), summer (June to August), and autumn (September to November) seasons. The calculation is based on the quantity of homes, hospitals, streetlights, and retail establishments in the vicinity. Specifically, while assessing the required electrical load, the anticipated operating times primarily at night of fifty residences, ten street lights, one clinic, and one business have been taken into account. The hourly and monthly load profiles are shown in Fig. 6. Based on this profile, the average annual power consumption for the village is estimated to be 437.56 kWh/day, with a peak demand of 90.12 kW [34].

**Table 3:** Estimated load need for the considered village.

Load type	Appliances	Power (W)	Nos.	Demand/	(Spring, Autumn,	Demand/	(Summer)
				(h/day)	(Winter) (Wh/day)	(h/day)	(Wh/day)
Residential	LED lamp	20	5	7	700	7	700
	TV	30-50	1	4	120	5	150
	Ceiling fan	30	2		-	24	1440
	Refrigerator	450	1	10	4500	24	10800
	Washing machine	325	1	1	325	1	325
	Rice cooker	700	1	3	2100	3	2100
	Water heater	2000	1	1	2000	-	-
	Electric kettle	1500	1	0.4	600	0.5	750

Total for 35 households					362075		569275

Community Clinic	LED lamp	20	4	14	1120	14	1120
	Ceiling fan	30	2	-		-1440	24
Road lights	Refrigerator	600	1	24	14400	24	14400
	LED lamp	70	10	13	9100	6	4200

Total Commercial					24620		21160

Shop	LED lamp	20	2	7	280	7	280
	Ceiling fan	30	1	-	-	15	450
	Refrigerator	450	2	24	10800	24	10800

Total					11080		11530

### 2.3 Component modeling and system configuration

**Figure 7:**
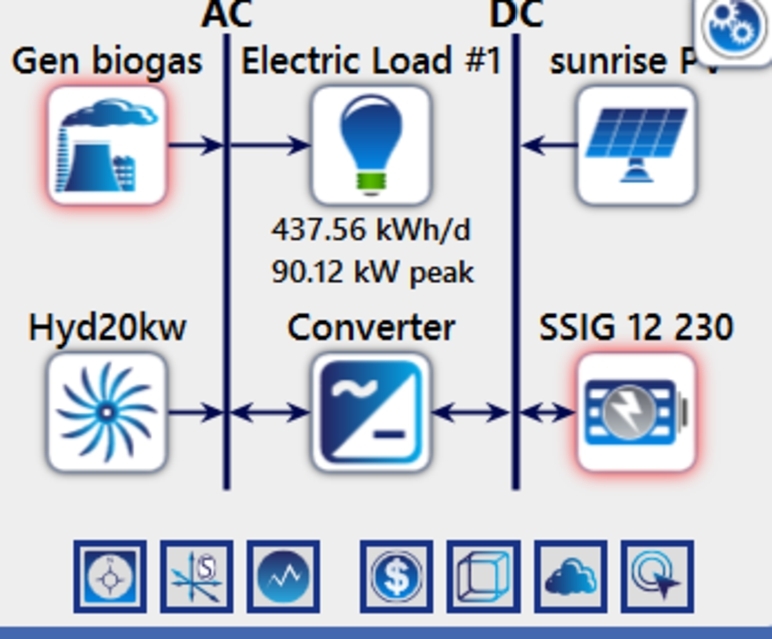
Schematic diagram of the PV-BG-HP-BESS hybrid system.

This hybrid power system consists of battery banks, generators, PV modules, direct current (DC) and alternating current (AC) buses, additional auxiliary equipment and load demand. The flowchart such system is shown in Fig. 7. In this system, PV, a converter and batteries are linked by a DC bus, whereas the DG, BG, converter, and electric load are connected via an AC bus.

#### 2.3.1 PV module

**Figure 8:**
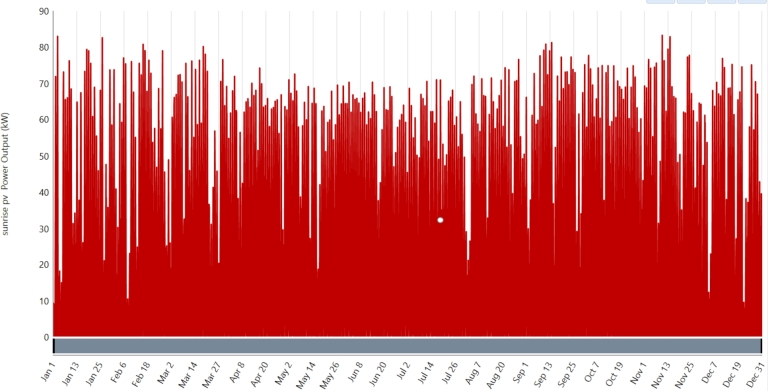
Photovoltaic output in kw.

The solar photovoltaic (PV) panel is the Sunrise SR-72M575NHLPro, offering a maximum power output of 575 Wp and a remarkable efficiency of 22.28%. With a maximum power voltage of 42.23 V and an open circuit voltage of 51.11 V, this panel is usable in temperatures ranging from -40 °C to 85 °C. This MBB-Mono-Crystalline Silicon panel possesses 144 cells arranged in 6 columns and 24 rows, with a short circuit current of 14.39 A and an ultimate electricity current of 13.62 A. The panel has dimensions of 2278 mm in height, 1133 mm in width, and 35 mm in depth, weighing 28 kg. Its robust structure and high coefficients of temperature ensure optimal performance in every type of environmental condition, with an open circuit voltage temperature coefficient of -0.249%/°C, the highest power temperature coefficient of -0.3%/°C, and a short circuit current temperature coefficient of 0.045%/°C. For the financial evaluation, both the replacement and capital costs are set at $200 per kWp, with a 25-year existence. Operating and maintenance (O&M) costs are insignificant, at $0 per kWp per year. The system's peak power ranges from 0 to 200 kWp at 10 kWp intervals, giving flexibility to meet changing energy needs. The solar electricity output is shown in Fig. 8. This complete parameter requirement ensures the selection of a reliable and effective PV panel for long-term power production. Table 4 [34] presents the parameter data of the PV modules at STC.

**Table 4:** PV module technical and financial information.

Parameter	Specification
Manufacturer	Sunrise SR-72M575NHLPro
Voltage at maximum power (V)	42.23
Operating temperature (^∘^C)	-40 to 85
Panel dimension (H/W/D) (mm)	2278113335 mm
Short circuit current (A)	14.39
No. of cells	144 (624)
Maximum power (Wp)	575
Weight (kg)	28 kg
Current at maximum power (A)	13.62
Open circuit voltage (V)	51.11
Cell type	MBB-Mono-Crystalline Silicon
Panel efficiency (%)	22.28
Temperature coefficient of open circuit voltage (%/°C)	-0.249
Temperature coefficient of maximum power (%/°C)	-0.3
Replacement cost ($/kWp)	200
Temperature coefficient of short circuit current (%/°C)	0.045
Capital cost ($/kWp)	200
Capacity (kWp)	0-200, 10 intervals
*O*&*M* cost ($/kWp/year)	0
Lifetime (years)	25

#### 2.3.2 Biogas generator

A key component of Pakistan's renewable energy landscape is biomass and biogas energy. To provide energy, the country uses a variety of biomass sources, such as wood biomass, agricultural waste, and animal dung. Interestingly, producing biogas from organic waste, such as cow and buffalo dung, is a common activity [36]. Agricultural wastes are converted into methane gas by anaerobic digestion processes, which is used for heating, cooking, and energy production. Pakistan strongly encourages the use of biogas technology to control trash and electrify rural areas that are prone to animal waste. In comparison to traditional energy sources, this sustainable energy strategy provides a greener, cleaner option. The estimated daily waste of a buffalo is 14 kilogram, while that of a cow is 10 kg. The estimated monthly average accessible biomass has a low thermal value (LHV) of 5.5 MJ/kg, a carbon content of 5%, a gasification ratio of 0.7, and an output of 2.85 tons per day. The estimated capital and replacement costs for the selected biogas system are $300 and $250 per kW, respectively, with an operating and maintenance cost of $0.05 per hour [39]. The system is expected to have a 20,000-hour lifespan. A list of the technical characteristics for the selected BG is provided in Table 5 [33].

**Table 5:** Parameter data assumed of the selected BG.

Parameter	Specification
Capital cost ($)	300
Minimum load ratio (%)	50
Heat recovery ratio (%)	0
Lifetime (hrs)	20,000
Replacement cost ($)	250
Capacity (kW)	0-50 , 75,100
*O*&*M* cost ($/hr)	0.040

**Figure 9:**
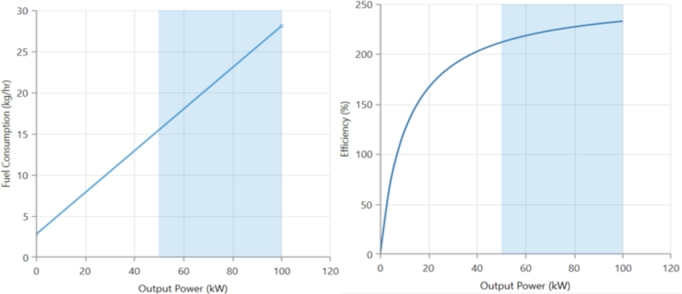
The chosen BG's fuel flow and efficiency curves.

**Figure 10:**
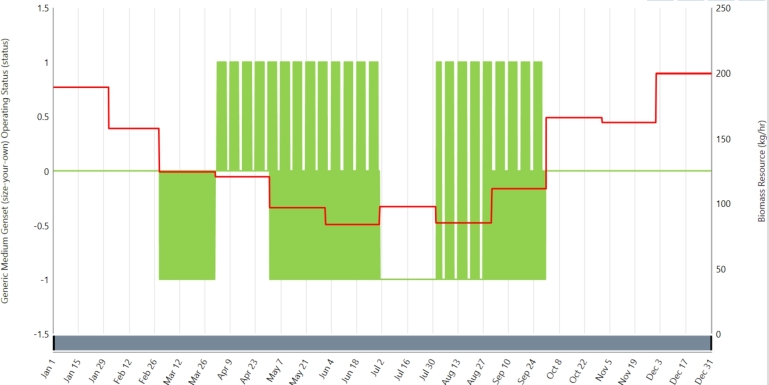
Biomass resource (kg/hr) and generic operating status.

Figs. 9(a) and 9(b) indicate the efficiency and fuel flow curves for the opted for BG, respectively. The fuel curve slope is found to be 2 kg/h/kW, the efficiency is around 30%, and the intercept coefficient is 0.1 kg/h. The biomass resource (kg/hr) and general operational condition are shown in Fig. 10.

#### 2.3.3 Batteries

**Figure 11:**
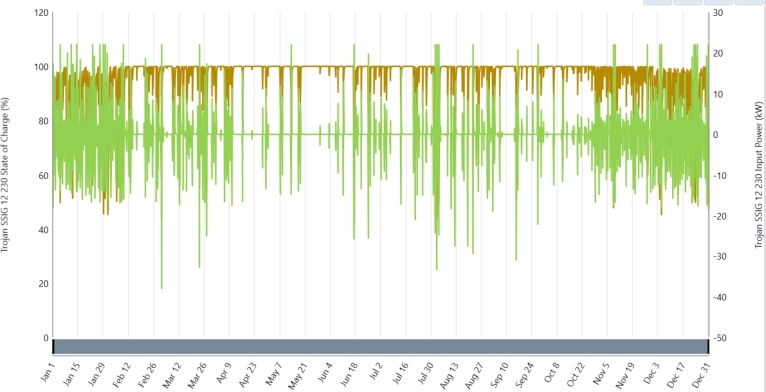
Battery 12 volt 230 Ah state of charge and input power.

Because of its safety features, steady performance, and low initial cost, lead-acid batteries are used in household energy storage [18], [37]. The study uses the Trojan SSIG 12 230 lead-acid battery, which boasts parameters like 230 ampere-hours, 12 volts, and a lifetime throughput of 2285 kilowatt-hours. Lead-acid batteries are recognized for their durability, reliability, and affordability in relation to other battery technologies. They have a long tradition of use and are popular in the retail sector. Still, they are larger and have a smaller energy density than newer battery technologies such as lithium-ion. Regardless of such drawbacks, lead-acid batteries are still an attractive option for applications that value cost-effectiveness and strength over size and capacity for energy. Fig. 11 shows the battery state of charge and input power. There is a 20% minimum permissible state of charge (SOC) and an 85% round-trip efficiency. It is expected that every battery will cost around 400 in capital, 300 in replacement, and 8 annually in operation and maintenance (O&M). Table 6 contains the battery's cost and technical specifications [42, 43].

**Table 6:** Details on the trojan SSIG 12 230 battery's specifications and price.

Parameter	Specification
Strings	0-50, 5 batteries intervals
Replacement cost ($)	300
Maximum capacity (Ah)	230
Minimum SOC (%)	20
Nominal capacity (kWh)	3
Capital cost ($)	400
Nominal voltage (V)	12
(*O*&*M*) cost ($/year)	8
Lifetime throughput (kWh)	2285
String size	5
Roundtrip efficiency (%)	85

#### 2.3.4 Hydropower

Hydroelectric power is an environmentally friendly source of electricity that releases no greenhouse gases during the process. It is also credible, as water flow can be ruled and modified to meet demand, making it a crucial component of a diverse energy combination. Hydropower systems are known for their reliability and long lifespan, making them a sustainable option for energy generation. The system's capital cost is $20,087, with a replacement cost of $5,120. Annual operation and maintenance (O&M) costs are estimated at $1,000 per year. a design flow rate of 0.06 cubic meters per second and with an available head of 1.67 meters, the system operates at an efficiency of 80%. This hydropower system is designed to last for 25 years, providing clean and efficient energy for a sustainable future. The technical and cost data is listed in Table 7.

**Table 7:** Turbine specification.

Parameter	Specification
Capital Cost	$20087
Replacement cost ($)	$5120
(*O*&*M*) Cost	$1000/year
Available Head	1.67 m
Design flow rate	0.06 m3/s
Efficiency	80%
Lifetime	25 Years

Innovatively, harnessing the hydropower potential along the BS link canal-1, which links the rivers Swat and Kabul, presents a compelling opportunity. Despite seasonal variations, with limited water flow to the BS link canal-2 in January, abundant water resources are generally available throughout the year. Mainly, the peak water flow occurs in June, July, and August, coinciding with the flooding of rivers due to glacier melting [35]. Hydropower systems harness the energy of flowing water to generate electricity. The water flow rate via a pipe may be calculated using the equation below.

(5) Q=AV

Equation (5) Q stands for the flow rate, v for the water's velocity, A for the pipe's cross-sectional area. The following steps are used to compute the turbine P's output shaft power:

(6) P=η×g×H×Q

**Figure 12:**
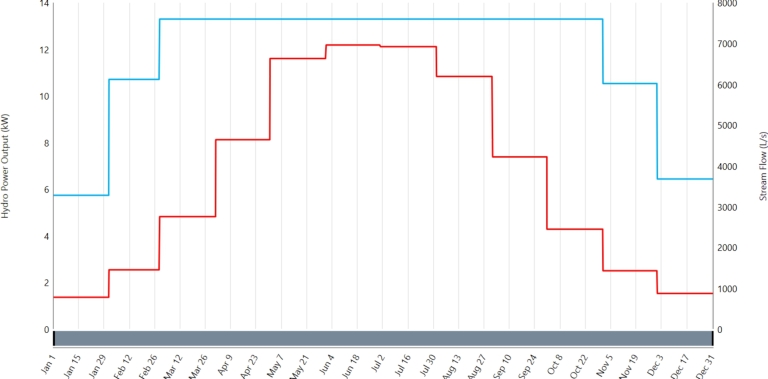
Hydro power output and stream flow.

In the above equation (6) the variables P (turbine output power), *η* (turbine efficiency), H (water head), Q (flow rate), and g (gravitational acceleration) are determined using the equation described before. Fig. 12 shows stream flow and hydropower output.

#### 2.3.5 Converter

Rectifiers, which convert AC energy to DC, and Inverters, which convert DC electric energy to AC, are the two main types of energy conversion devices used in hybrid power systems [38]. The analysis makes the premise that the inverter has a 15-year service life and an efficiency of 95% (see Table 8).

**Table 8:** Details of the chosen converter.

Parameter	Specification
Efficiency (%)	95
Capital cost ($/kW)	350
Size (kW)	0-80, 10 intervals
(*O*&*M*) cost ($/year)	20
Replacement cost ($/kW)	300
Lifetime (years)	15

#### 2.3.6 Dispatch strategy (DS)

When renewable energy sources alone are unable to fulfill load demand, a dispatch strategy (DS) is a set of guidelines that directs the operation of generators and energy storage devices. Two main dispatch algorithms are used in multi-energy power generating systems: cyclic charging (CC) and load following (LF) and [40], [41]. Diesel generators (DGs) under the LF approach run at full rated capacity in order to directly supply the load and charge the batteries. DGs can power the load automatically and charge the batteries at the same time using this method. On the other hand, the DGs in the CC method are used to charge the batteries rather than power the load directly. With this method, the load is not powered only by the DGs. The ideal analytical method is established by considering the dispatch techniques reported in the literature [40], [41], [42].

## 3 Result and discussion

The plan of fortification of a hybrid energy system is quite promising strategy, which ensures optimal values for several criteria including direct and indirect profits, decrease of volumes of damaging gaseous emissions, flood risks management, and electricity production. It becomes apparent how the system applies renewable energy to lower costs achieved by solar photovoltaic (PV) panels, Biogas (BG) producers, hydro power (HP), and battery storage. It reduces the use of fossil energy sources and helps decrease hazardous gas emissions, as well as reduces the degree of impact on the natural environment, by using hydropower we are also able to manage flood catastrophe because it controls the flow of water. The system has long-term economic value due to the uniform production of power and cheaper operational expenses. To sum up, the suggested hybrid energy system is effective and capable to meet energy demands as well as address ecological and financial challenges.

The usage of the biogas generator in the simulation and what season it is in changes in order to accommodate the demand from the customers and the available resources. Namely, when solar radiation and water resources are inexpensive from March to September, photovoltaic (PV) panels as well as hydropower meet the electricity needs. On the other hand, in the case of scarce solar and water resources for the remaining part of the year, the biogas generator is employed to meet the required electric power. This kind of flexibility allows RE sources such as PV and hydropower to be consumed to the last drop during summer. Thus, to cheaply use the natural resources, the biogas generator is switched off during the period, May-August when the PV resources and water flow are substantial. However, again, it can be utilized in December and January, if the stock of such resources is low. This method ensures that renewable energy sources are prioritized during peak supply while the biogas generator operates as a reliable backup for less optimal requirements. In summary, this method strengthens the hybrid energy system's reliability and sustainability by optimizing the everyday use of green energy sources.

### 3.1 Cost minimization

**Figure 13:**
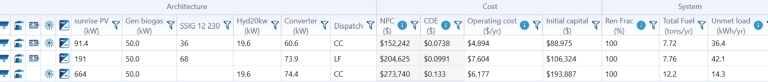
Categorized system configuration schemes.

PV-BG-HP-BESS, the suggested hybrid energy system, operates as planned to minimize costs. The community in Tangi has an average annual global solar radiation of 4.12 kWh/m2/day and 2.85 t/d of biomass available each year at a cost of less than 20 per ton. The ideal setup is shown in Fig. 13 and consists of a 50 kW biogas generator, 36 batteries, a 91.4 kW photovoltaic (PV) system, and 19 kW of hydropower. Using the cc approach, this arrangement yields a Cost of Energy (COE) of $0.0738/kWh and a Net Present Cost (NPC) of $152,242. Nevertheless, the NPC climbs to $204,625 and the COE to $0.0991/kWh when hydropower is disregarded, suggesting that hydropower is essential to the system's economic sustainability. This ideal system has an annual operating cost (OC) of $4,894 and an initial capital cost (IC) of $88,975. Furthermore, as shown in Fig. 13, the system has a 100% Renewable Fraction (RF), demonstrating its ecologically benign and sustainable characteristics. [Li C, Zhang L, Qiu F, Fu R]. When using the Load Following (LF) method, the PV/BG/battery hybrid system with a 400 kWp PV array, 100 kW BG, 400 batteries, and a 200 kW converter proves to be the most economical choice, with the lowest Net Present Cost (NPC) of $1,808,992 and Cost of Energy (COE) of 0.24 $/kWh. However, a comparable study has already been conducted in the literature, for example, in which the DG system employing the cyclic charging (CC) strategy is economically unfeasible, with the highest NPC of $5,419,563 and COE of 0.692 $/kWh [18].

### 3.2 Sensitivity analysis

**Figure 14:**
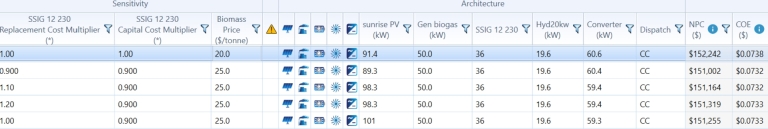
Snsitivity analysis of proposed hybrid energy system.

Sensitivity analysis is an effective method to analyze a model or system's flexibility and resilience to changes in its input criteria. In the revised version, Fig. 14, to examine the effects of biomass and battery price increases on the optimal hybrid power system, a sensitivity analysis was conducted with biomass prices ranging from $0 to $100 per ton, with increments of $20 per ton. Similarly, the battery capital cost was obtained by varying it near a base value of $400 using a multiplier of 0.9, 1, 1.1, and 1.2. This means battery costs of $360, $400, $440, and $480, respectively. Biomass prices are also estimated to be $25, $30, and $35 per ton. When battery capital costs were analyzed, it was found that as battery costs developed, so did COE and NPC. The PV/BG/battery hybrid system stays optimal in the battery capital cost range of $360 to $480. Additionally, the PV/BG/battery system is the better option for biomass prices between $25 and $35 per ton, illustrating its flexibility within cost levels. Similarly, the battery capital cost was obtained by varying it near a base value of $400 using a multiplier of 0.9, 1, 1.1, and 1.2. This means battery costs of $360, $400, $440, and $480, respectively. Biomass prices are also estimated to be $25, $30, and $35 per ton.

### 3.3 Release of toxic gases

The research evaluated biogas and crambe biodiesel (B0-B100) as alternative fuel sources for engines. The dual mode (biogas) provided 17% greater power with less fuel usage than the usual mode (biodiesel), according to the results. In the dual mode, nitrogen oxide emissions rose with a larger biodiesel percentage but reduced with biogas. As biodiesel content increased, carbon monoxide emissions dropped. All things considered, partly replacing diesel in dual fuel engines with biogas and crude biodiesel is an effective method [39].

The paper explains how a water turbine (hydro), battery energy storage system (BESS), photovoltaic (PV), and diesel generator (D) are integrated into the hybrid energy management system. The diesel generator is one of the generators that are used by incorporating several generators power in order to enhance the improvement of economic as well as industrial benefits and the protection of the environment. However, the emphasis is placed on restriction of its usage because of the negative influence of diesel generators on the environment, for example, emissions of toxic gases [43].

An assessment of the proposed system means that there will be reduced burning of noxious gases and, hence, cleaning of the air, thereby directly enhancing public health through the prevention of, among others, heart and lung diseases. In addition, the reduction of various greenhouse gases, including CO2 and methane, is unavoidable if climate change is to be arrested so that the societies temperature is not raised, thus comprehensively countering any extreme weather, including storms, sea level rise, and decline in biodiversities. These environmental benefits not only help improve the quality of the present world but also create a better world to dwell in for the generations yet to come.

Thus, as we have included the renewal of energy sources, many different approaches can be implemented to bring down emissions even more. These include utilizing renewable power like LED lighting and high-efficiency machines to help minimize energy usage. Reducing waste and measures like composting and recycling could help decrease the amount of methane that is generated from garbage disposal. Furthermore, CCS technologies help reduce the manufacturers CO2 emissions by recording them and storing them underground to prevent their release into the atmosphere. As identified as one of the best hybrid energy systems, the inclusion of photovoltaic, biogas, hydro, and battery proves environmentally friendly and sustainable to the rural regions of Pakistan. Toxic gas and pollutant emissions would significantly increase if a diesel generator were used in place of a biogas generator. For example, the carbon dioxide emissions from a similar 2 MW diesel generator would be around 5,419,563 kg/yr more than 2,000 times more than from the biogas generator. The diesel generator would contribute to air pollution and environmental deterioration by releasing more carbon monoxide, sulfur dioxide, and nitrogen dioxide into the atmosphere. Thus, the use of the biogas generator in the hybrid system lessens the environmental issues associated with using standard diesel generators in addition to enabling continuously sustainable power from electricity to be acquired. The comparison of diesel and biogas generators is shown in Table 9.

**Table 9:** Comparison of biogas and diesel generator emissions.

Quantity	Emissions of biogas gen	Emissions of diesel gen	Unit
Carbon dioxide	1.29	2,522	Kg/yr
Carbon monoxide	0.148	16.2	Kg/yr
Sulfur dioxide	0	6.17	Kg/yr
Nitrogen dioxide	0.0118	12.8	Kg/yr

### 3.4 Flood disaster

The suggested improvement of the incorporation of a flood disaster controller, which is designed for medium canals, into the described hybrid energy system can help to substantially streamline flood control measures in the rural areas of Pakistan. The controller would use the energy produced by the photovoltaic panels, biogas, hydro, and battery to operate a number of sensors and actuators that are to be planted along the canal.

The controller continuously receives data from sensors monitoring the water level and flow in the canal. When sensors detect high water levels, they activate gates or valves to regulate water flow and prevent floods. This paper has shown that utilizing the flood disaster controller in the hybrid energy system can benefit rural communities through better flood control, less frequent destructive agriculture and infrastructure damages, and greater tolerance to climate change. The system incorporates renewable energy technologies like photovoltaic panels and biogas while symbolizing the means to unique local issues, hence improving services to the communities served.

### 3.5 Economic benefits

The following are some of the financial benefits of the hybrid energy system, which has PV plates, methane digesters, hydropower turbines, and batteries in the rural areas of Pakistan. First, with the ability to gradually decrease the reliance on expensive fossil fuels, it also slows the growth of operating costs over time. Pakistan has ample sunlight, which makes photovoltaic (PV) panels a stable power-producing source that provides electricity & cuts electricity costs even in rural areas. Through the use of organic waste, biogas digesters produce biogas for cooking and heating instead of the standard wood and Gas cylinder. Furthermore, the applications of hydroelectric turbines are easy to maintain and are counted on for a sustainable energy source to be generated through the flow of water. Battery storage systems stabilize energy and avoid costly grid outages by storing energy in excess during some periods for use during other periods of high usage or low production. Contributes to the sustainable development of society while significantly increasing the rate of economic growth through reduction of energy costs, creation of employment opportunities for citizens, and enhanced energy security.

In the framework of energy sustainability, the definition of “energy democratization” can be considered modern and progressive. This embraces the devolution of energy generation, delivery, and consumption, thus allowing the communities to be involved actively and benefit from the energy transition. The given plan of this hybrid energy system fits perfectly into the category of energy democratization because it is based on the use of many types of renewables, including solar, biogas, hydropower, and battery energy storage in different villages in Pakistan. Self-organization of local communities with regard to energy resources increases their autonomy and protects them from disturbances. Besides, it entails an increased engagement in societal and environmental matters including grassroots renewable energy invention and enterprise, which also fuels economic development. As such, energy democratization is an approach to bringing into realization the progressive social processes of justice and sustainability guaranteed for the improvement of the lot of all individuals in society.

### 3.6 Advantage of the proposed hybrid system

The most suitable as well as inexpensive hybrid energy systems for Pakistans rural areas include photovoltaic (PV), biogas, hydro, batteries, and the following are some advantages. First, this system reduces the dependency on expensive as well as polluting sources of energy, such as fossil fuels, through the provision of a reliable source of electricity. This assures the availability of electricity for the system and other electrical gadgets despite accessing regions that are far from the power generating companies by adopting the use of hydro-turbines to generate electricity from water, fermenting organic waste products to create biogas and using the PV panels to generate power from solar energy. The detailed perks of biogas generators and the demerits of diesel generators are presented in Table 10 in the following manner. Similarly, battery storage in the integration increases energy flexibility and saves expensive system disruption energy for high demand or low generation. Also, through cutting down on emissions of hazardous gases, preventing air pollution as well and mitigating the effects of climate change, the hybrid system is noted to have a positive environmental impact. Thus, this study finds that Pakistans issues with rural electrification may be solved with an efficient combination of renewable energy sources in what could be called a workable and sustainable hybrid energy system that will contribute to the achievement of environmental goals, energy security, and economic growth.

**Table 10:** Benefits of biogas generators and drawbacks of diesel generators.

Biogas Generator	Diesel Generator
Renewable energy source	Non-renewable energy source
Production from organic waste reduces landfill	Emits greenhouse gases and pollutants
Lower operating costs	Higher operating costs
Can be produced locally, reducing dependence on imported fuels	Reliance on imported fossil fuels
Can provide a stable and constant energy supply	Energy supply dependent on fuel availability and logistics

### 3.7 Overall power generation of the proposed hybrid energy system PV-BG-HP-BESS

**Figure 15:**
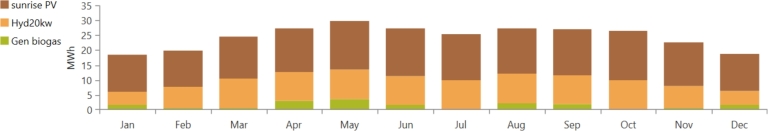
Monthly electric production of PV, HP and BG.

**Figure 16:**
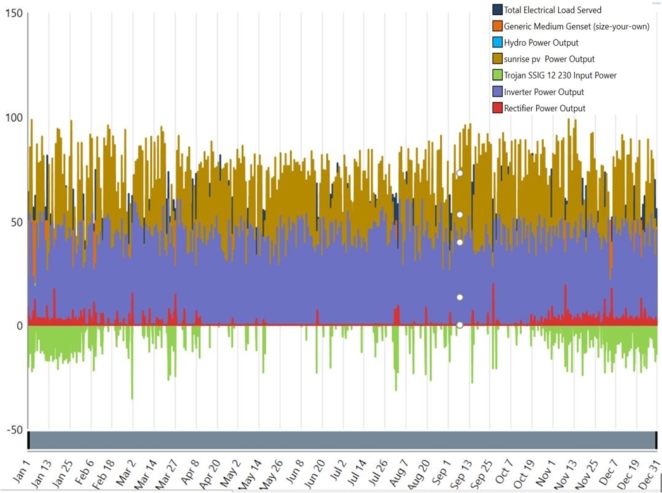
Uniform power generation of the proposed hybrid energy system.

Fig. 16 shows the consistent power generation of the proposed hybrid energy system, total electric load served, and inverter power output through year PV-BG-HP-BESS. Amazingly, the system produces 294,782 kWh of electricity annually. In the proposed system, PV contributes the most, producing 175,028 kWh (59.4%), while BG generates the least, producing 17,746 kWh (6.02%), and hydropower produces 102,007 kWh (34.6%). The ideal system has an excess of 129,709 kWh/year (44%) of electricity, an unmet electric load of 36.4 kWh/year (0.0228%), and a capacity shortage of 150 kWh/year (0.09%). The average monthly power output for the ideal PV-BG-H]P-BESS system is shown in Fig. 15. Also, January through May are the months with the highest PV power generation, which is explained by the sun's highest radiation levels during these months. October through December have lower solar radiation levels, which explains the lower PV power generation.

The overall production in Fig. 16 of the proposed hybrid energy system is given in the below.

## 4 Conclusion

Using the HOMER simulation approach, the research carried out a thorough examination of biogas, a hybrid photovoltaic, hydro, and battery sustainable energy system for a hamlet in Charsadda, Pakistan. The best possible system design showed improved economic and environmental performance. It included 36 batteries, a 50 kW biogas engine, 19.6 kW hydropower, and 91.4 kWp PV modules. The system demonstrated its superior cost-effectiveness with an NPC of $152,242 and a COE of $0.0738/kWh, underscoring the role of hydropower in augmenting economic viability. The system's environmental friendliness was highlighted by the considerable reduction in pollutant emissions compared to diesel generators when PV and BG were integrated.

In general, the hybrid energy system helps rural communities by lowering their dependency on expensive fossil fuels and giving them a dependable and sustainable energy supply. It promotes sustainable development and gives communities the ability to control their energy resources, making it a prime example of the idea of energy democratization. This system offers Pakistan's rural electrification and energy sustainability a modern, forward-thinking solution by fusing renewable energy sources with state-of-the-art technology. The proposed system provides significant environmental advantages. Table 9 is essential regarding pollutant emissions and economic benefits compared with other systems in the literature. More specifically, the proposed system's net cost of production per kilowatt of energy dominates other systems in the literature, as given in Table 1.

Additionally, using multiple renewable energy sources increases reliability, facilitating a consistent power supply for remote areas. Possible drawbacks include limited scalability due to the Charsadda-specific setup and reliance on good environmental conditions for peak performance. The system's complexity during implementation may also present challenges that require careful preparation and abilities for successful installation.

### 4.1 Limitatation and future directions

There are a few limitations to the proposed work on optimal and sustainable hybrid energy systems in the rural region of Pakistan. The main restriction is that current data on the local energy demand and the environment need to be revised and refined for the sake of the models. The areas for further research and development should relate to the improvement of innovative grid technologies for more effective energy management. Also, there are methods of financing that can be applied to the proposed system, together with corresponding regulations for the systems profitability. Additionally, it is widespread to develop the system environmental impact evaluation on the various chemicals and the different mitigation approaches. Last but not least, adding energy storage systems, like CAES or LAES, to the power system might enhance its availability.

### 4.2 Significance of the proposed work

It forms an innovative solution with different types of hybrids (PV-BG-HP-BESS) consisting of photovoltaic, biogas, hydropower, and battery storage. The technology has a creative design that empowers an efficient generation and utilization of energy that lasts for an enormous amount of time, thus reducing emissions of carbon. The suggested system not only addresses the problems of energy scarcity but also provides a model for integrative renewable and cheap power in the rural world settings.

I. Addressing energy poverty: Thus, according to the findings of this article, individuals should blend renewable energy sources (biogas, solar, and hydroelectric) has extended the prospects of eradicating energy poverty in the rural areas Pakistan.
II. Flexibility and reliability: The incorporation of varied energy sources forms a hybrid system, which can improve variable energy efficiency. This helps guarantee a very stable power supply system, which would be essential in areas where there are frequent or constant power blackouts. The Unique feature of a hybrid system is the flexibility of incorporation of multiple renewable energy sources, which significantly minimizes the use of fossil fuels. This not only helps to reduce the greenhouse effect but also helps to protect the environment, which is of utmost importance to green gurus.
III. Seasonal Variation Mitigation: The system design considers variations in solar and hydroelectric power generation depending on the seasons to maintain a consistent energy supply for the year.
IV. Optimization and efficiency: This article examines optimizing methods in developing hybrid energy systems to ensure optimal sizing and arrangement for maximum economic efficiency. Despite the initial high cost, the hybrid system offers substantial long-term economic benefits. These include minimized operating and maintenance costs and the potential for government assistance, ensuring financial security, and appealing to the financial interests of policymakers and government officials.
V. Regional and global impact: Executing this hybrid energy system has the opportunity to significantly enhance the lives of local populations, especially in areas undergoing an energy crisis, such as Pakistan's rural areas. It corresponds with international efforts to reduce climate change and decrease greenhouse gas emissions.

## Funding

The authors express their gratitude to Princess Nourah bint Abdulrahman University Researchers Supporting Project Number (PNURSP2024R17), 10.13039/501100004242Princess Nourah Bint Abdulrahman University, Riyadh, Saudi Arabia. This work was supported by the Deanship of Scientific Research, Vice Presidency for Graduate Studies and Scientific Research, 10.13039/501100020912King Faisal University, Saudi Arabia (KFU241446).

## Authors' contributions

All authors participated equally in the preparation of this manuscript. All authors verified the final version of this manuscript. All authors contributed equally to this work. All authors read and approved the final manuscript.

## Use of artificial intelligence (AI) tools declaration

The authors declare they have not used AI tools in the creation of this article.

## CRediT authorship contribution statement

**Safyan Mukhtar:** Funding acquisition. **Shakoor Muhammad:** Software, Investigation, Formal analysis, Conceptualization. **Haifa A. Alyousef:** Validation, Supervision. **Wajid Khan:** Writing – original draft, Formal analysis. **Rasool Shah:** Visualization, Validation. **Samir A. El-Tantawy:** Validation, Funding acquisition.

## Declaration of Competing Interest

All the authors declare that they have no conflict of interest.

## Data Availability

Data sharing does not apply to this article as no data sets were generated or analyzed during the current study.
